# Mean Platelet Volume and Immature Platelet Fraction in Autoimmune Disorders

**DOI:** 10.3389/fmed.2017.00146

**Published:** 2017-09-06

**Authors:** Deonilson Schmoeller, Maria Mercedes Picarelli, Terezinha Paz Munhoz, Carlos Eduardo Poli de Figueiredo, Henrique Luiz Staub

**Affiliations:** ^1^Rheumatology Department, Saint Lucas Hospital, Pontifical Catholic University of Rio Grande do Sul (PUCRS), Porto Alegre, Brazil; ^2^Pathology Laboratory, Saint Lucas Hospital, Pontifical Catholic University of Rio Grande do Sul (PUCRS), Porto Alegre, Brazil; ^3^Nephrology Department, Saint Lucas Hospital, Pontifical Catholic University of Rio Grande do Sul (PUCRS), Porto Alegre, Brazil

**Keywords:** mean platelet volume, immature platelet fraction, autoimmune diseases, platelet size, platelet volume

## Abstract

Mean platelet volume (MPV), measured using automated blood analysers, has been appraised as a potential biomarker in cardiovascular disease, diabetes mellitus, and cancer. The test, a useful tool in differentiation of thrombocytopenic states, has now been carried out for autoimmune disorders, but data are yet scarce. Controversial results have been obtained in systemic and organ-specific autoimmune disorders. Another test, the immature platelet fraction (IPF) reflects the amount of young, reticulated platelets. IPF is calculated by automated hematology analysis or flow cytometry, and it is usually high in patients with rapid platelet destruction. For both MPV and IPF, standardization of cutoff is a major need. In this review, we focus the current applicability of MPV and IPF as biomarkers in patients with autoimmune diseases.

## Introduction

Platelets are intriguing and complex cells. Approximately one trillion platelets circulate in blood to provide vascular regulation. Nowadays, the biological functions of platelets are considered to be far beyond hemostasis and thrombosis. Platelets have also been linked to inflammation, atherosclerosis, autoimmunity, and tumor immunology (1).

Platelet receptors such as CD40, GPIb/IX/V, and selectins have all been implicated in perpetuation of atherosclerosis, rheumatoid arthritis (RA), and tumors. Knowingly, platelets can recognize bacteria and attract immune cells to inflammatory sites. Also, they release granular contents (growth factors) active in wound repair (2).

In autoimmune diseases such as systemic lupus erythematosus (SLE), immune complexes activate platelets by interacting with Fc receptors; in RA, the platelet is a well-known source of prostaglandins within the inflamed synovium. IL-1-containing platelet-derived vesicles are abundant in synovial fluid and stimulate synovial fibroblast to produce inflammatory mediators. Moreover, serotonin released by platelets enhances vascular permeability within the inflamed synovium (3).

Platelet size heterogeneity is mostly determined by variations in territory growth and demarcation, but not accurately upon aging in circulation. Size correlates with cell activity and can be assessed by volume indices (4). Among them, the mean platelet volume (MPV), routinely measured in blood cell count, and the immature platelet fraction (IPF) are tests of current interest (5).

In this review, we will be focusing on the “non-hemostatic” functions of platelets in rheumatic and non-rheumatic autoimmune disorders. We will particularly approach the clinical applicability of MPV and IPF in such a context.

## MPV: General Aspects

Mean platelet volume, normally measured using automated blood analysers, reflects the average size of platelets in circulation. It is meant to show the relationship between platelet synthesis in bone marrow and cell destruction. A normal MPV has a range of 7.5–11.5 fL. MPV correlates with platelet function and may be more sensitive than platelet count as a biomarker in a variety of disorders. It is also regarded as a useful surrogate marker of platelet activation or reactivity (4, 5). Clinical utility of MPV has been a matter of debate for the last few years. MPV cutoff has not been fully validated so far, and standardization is a major need.

The test is particularly useful in patients with thrombocytopenia and thrombocytosis. Thrombocytopenia with high MPV is seen in immune thrombocytopenic purpura (ITP), disseminated intravascular coagulation (DIC), sepsis, and preeclampsia. Thrombocytopenia with low MPV is typical of patients with low platelet production, i.e., aplastic anemia. In patients with high platelet counts, a high MPV is suggestive of primary thrombocytosis, while a low MPV characterizes a reactive thrombocytosis, seen in infection, inflammation, or malignancy (5).

There has also been a body of interest for MPV in patients with normal platelet counts. In a 2010 meta-analysis, an elevated MPV associated with acute myocardial infarction (AMI), mortality after AMI, as well as restenosis following coronary angioplasty. The test might behave as a prognostic biomarker in individuals with cardiovascular disease (6). MPV is augmented in patients with type 2 diabetes mellitus (DM) and might associate with risk of cardiovascular events in this population (7).

Mean platelet volume level was significantly higher in malignant tumors than in healthy subjects and decreased with therapy, according to a recent meta-analysis (8). Clinical utilization of MPV in cardiovascular disease, DM, and malignancy is open to discussion.

## MPV and Rheumatic Diseases

A low MPV is usually related to inflammatory states. A recent study correlated MPV with inflammatory and disease indexes in rheumatic disorders. An inverse correlation of MPV with erythrocyte sedimentation rate (ESR), C-reactive protein (CRP), and DAS-28 was found in RA patients. MPV correlated negatively with both the BASDAI in ankylosing spondylitis (AS) and with CRP in psoriatic patients. An inverse correlation of MPV with ESR was also seen in lupus patients. Overall, a low MPV level surrogated inflammatory states in a large number of rheumatic patients. The authors suggested that MPV works as a negative marker in these patients (9).

There has been a recent interest in MPV levels in patients with SLE. A Mexican study accounted for lower MPV levels in active lupus patients as compared to those with inactive disease. At a cutoff level of 8.32 fL, MPV sensitivity and specificity for detecting active disease were 86 and 41%, respectively. Of note, a positive correlation of MPV with albumin levels was documented (10). Another group of authors reported lower MPV levels in patients with active lupus arthritis in comparison to SLE patients in remission or healthy controls (11).

In 20 patients with juvenile SLE, differently, MPV was found to be higher in active than inactive patients or controls. A cutoff level of 8.4 fL was predictive for disease activation (sensitivity 75%, specificity 90%). MPV was positively correlated with SLEDAI, CRP and negatively correlated with C3. The authors concluded that MPV can be an early marker of SLE reactivation in children (12). As seen, data on MPV levels in SLE patients are conflicting in juvenile and adult populations; newer, prospective, studies are warranted to evaluate this potential marker in SLE (12).

Few, but interesting, data concern MPV in patients with antiphospholipid syndrome (APS), a thrombotic diathesis typical of young individuals. Twenty-two patients with APS were assessed during a thrombotic event and 3 months ahead. Of note, MPV was significantly high during the event and normalized 3 months later with treatment (13). In another study, 70 APS patients had MPV compared to healthy controls. MPV was higher in patients, and levels were particularly elevated in those with triple positivity for antiphospholipid antibodies. Of major interest, multivariate analysis demonstrated that an MPV above 7.4 fL significantly predicted thrombosis recurrence in APS patients (14). These data on APS suggest that a high MPV relates to vascular obstruction.

We also reviewed data regarding MPV utilization in RA patients. Yet in 1988, a significant inverse relationship of MPV with platelet count was observed in RA subjects (15). In a recent study including 100 RA subjects and 100 controls, MPV (cutoff of 10.4) was significantly higher in RA. MPV was similar to ESR and CRP to disclose inflammatory activity, but the test did not correlate with pain and disease activity score of 28 joints (DAS28) (16).

In contrast, MPV values were found to be lower in active RA patients than inactive, according to a 2015 report (17). Of note, TNF blockers promoted a significant increase in MPV levels after 3 months of therapy, according to a 2010 description (18). Yazici et al. reported higher MPV in RA patients than controls; the tested significantly correlated with ESR, CRP, and DAS28. Both anti-TNF-alpha and conventional therapy decreased MPV and other markers of inflammation (19).

Gasparyan et al., using a 10.7 fL cutoff, documented higher values of MPV in RA patients than controls. In RA patients, hypertension was associated with high levels of MPV, suggesting that this platelet indice eventually reflects an enhanced vascular risk (20). A 2008 study accounted for lower levels of MPV in RA and AS patients with active disease in comparison to controls. Levels increased with treatment (21). As seen, data concerning MPV as a biomarker in adult RA patients are far from homogeneous.

In one of the few reports looking at the biological behavior of MPV in juvenile idiopathic arthritis (JIA), 115 patients and 64 controls were evaluated. An augmented MPV was demonstrated in active disease as compared to inactive disease and controls. Regular therapy diminished platelet activation (22). In a survey of 55 patients with JIA, MPV measured after 2 months of treatment did not significantly vary in comparison to results in the acute phase (23).

Reports on MPV in patients with AS have been increasingly available. A very recent study disclosed that MPV is significantly higher in AS patients than in the controls; however, no correlation of MPV with disease activity index (BASDAI) was found (24). Data from Bozan et al., in turn, showed no difference in MPV values of AS patients and healthy controls (25). In a report where AS patients were stratified in osteopenic or not, MPV values were higher in the former, suggesting a role for MPV in bone loss (26). Data from Yazici et al. showed that MPV was significantly higher in patients with AS than controls; values were reduced by conventional or anti-TNF therapy. Also, a positive correlation of MPV with BASDAI was observed (27).

Reports of MPV in psoriatic arthritis are sporadic. According to a 2010 study, MPV levels were higher in patients with psoriasis and psoriatic arthritis than controls. MPV may be a marker of severe psoriasis. Also, the authors suggested that increased platelet activation might associate with cardiovascular risk in these patients (28).

In an isolated report in the issue, Soydinc et al. evaluated MPV in 76 patients with scleroderma and 45 healthy controls. MPV was significantly higher in patients, particularly in those with cardiopathy, digital ulcers, and gangrene. Increased MPV might be a marker of vascular disease in scleroderma (29).

As compared to a similar number of controls, MPV of 53 patients with acute rheumatic carditis did not significantly differ (30). A Chinese study with a large survey of subjects with Kawasaki disease (KD) showed lower MPV of patients as compared to controls (31). In turn, a previous meta-analysis had shown that the levels of MPV, among various other markers, were significantly higher in KD patients with coronary disease than those without coronary involvement (32). The precise role of MPV as a marker of KD (with or without coronaritis) is a pending issue.

## MPV and Other Autoimmune Conditions

The MPV has been investigated as a potential biomarker in a variety of non-rheumatic autoimmune disorders. It seems a hot topic at the moment, but most studies have low samples and lack a greater level of evidence. In Crohn’s disease (CD), for instance, data are unclear. Even though a decrease in MPV has been documented in patients as compared to healthy controls, the test was not able to distinguish active from inactive CD patients, according to a 2012 report (33).

In patients with ulcerative colitis (UC), a similar decrease in MPV was noted as compared with healthy controls. As opposed to CD patients, MPV of active UC patients was significantly lower than that of inactive UC patients. For determining active UC activity, sensitivity was 67% and specificity was 73%. Of note, a negative correlation of MPV with endoscopic activity index was described (34).

Pediatric patients with type 1 DM were found to show high MPV, and this finding might relate to future cardiovascular events (35). Another study accounted for raised MPV in type 1 DM, but without concomitant hyperaggregability (36).

Idiopathic sudden hearing loss (ISHL) may have an autoimmune origin. MPV was significantly higher in patients with ISHL than healthy controls, leading to the hypothesis that this syndrome is also characterized by ischemic events (37). Very recently, decreasing MPV levels were documented in individuals with subjective tinnitus, also potentially an autoimmune disorder (38).

In a study featuring 19 patients with bullous pemphigoid, high levels of MPV and eosinophil levels were detected. MPV may work as an indicator of cardiovascular risk in these patients, but this is yet to be proved (39). Chronic urticaria frequently associates with autoimmune systemic disorders such as SLE and RA and can be itself an autoimmune condition. Higher MPV was described in patients with this dermatological disorder as compared to controls (40).

## IPF: General Aspects

The IPF represents the percentage of circulating platelets which still retain RNA. It is a modern parameter measuring young, reticulated, platelets in peripheral blood. IPF is a rapid and inexpensive automated biomarker for etiology of thrombocytopenia. It is usually determined by flow cytometry or hematology analysers. According to a recent study in a large Danish population, reference interval for IPF as determined by hematology analyzer was 1.3–9% (41).

Immature platelet fraction cutoff is not a closed question; however, Korean data accounted for reference intervals of 0.5–3.2% in men and 0.4–3.0% in women, respectively (42). Standardization of IPF cutoff levels is a clear need.

The IPF is usually high in conditions where rapid platelet destruction is observed (ITP, as mentioned, but also thrombotic thrombocytopenic purpura and DIC). In thrombocytopenic disorders with low bone marrow activity or suppression, IPF is generally low (43).

The test has been increasingly ascertained in other clinical conditions. For instance, IPF levels, as well as MPV counts, were significantly higher in patients with gestational hypertensive disorders than controls, according to data from our Hospital (44). High IPF levels were documented in individuals with severe sepsis/septic shock, being correlated with severity scores. IPF might be considered as a biomarker of sepsis (45). The biological role of IPF in autoimmune conditions, ITP apart, is an interesting issue yet to be explored.

## IPF in Autoimmune Diseases

In practical terms, ITP is the only autoimmune disease whereby the IPF has been extensively evaluated. Very recent data from a large population of patients with thrombocytopenia disclosed that Sysmex-measured IPF values were well higher in ITP than non-ITP patients (16.3 versus 7.6%). Sensitivity of IPF as biomarker of ITP was 85.7% (46). In childhood, a cutoff value of 9.4% was practically diagnostic for ITP, with a sensitivity of 88% and a specificity of 85.7% (47).

According to a recent report, the test, although useful for screening of macrothrombocytopenia, can be influenced by platelet size (48). Both IPF and platelet-associated antibody were valuable in differential diagnosis of ITP, but the former showed a better performance (49). Interestingly, low IPF levels were seen in patients with refractory thrombotic microangiopathic hemolytic anemia. Serial IPF may guide therapy in these patients (50).

## Limitations

There are some limitations about MPV and IPF assessments. Preanalytical drawbacks are important and can affect results. For example, the method of venipuncture and the degree of accuracy of filling and gently mixing the sampling tubes can cause platelet activation (51). Other methodological interference can succeed secondary to the choice of standard anticoagulant, time interval of the measurement, specimen transport, and best storage temperature of sample tubes. Lancé et al. (51), in a recent review of these techniques, referred the lack of literature about it. There are also different techniques in assessing platelet counting and MPV assessment: impedance counting, optical light scatter counting, and immunological flow cytometry techniques. All these elements indicate a need for standardization of the MPV and IPF methods, to achieve the minimum possible interference on the results.

## Conclusion

- Mean platelet volume, a rapid and practical test available in blood cell count, represents platelet reactivity. The test is useful to discriminate causes of thrombocytopenia.
- In patients with autoimmune rheumatic disorders, the usefulness of MVP as a biomarker remains to be defined. Discrepant results have been reported in SLE and RA. In non-rheumatic or organ-specific disorders, data are again heterogeneous.
- Immature platelet fraction reflects the amount of young platelets in circulation. Apart from ITP, where it is confirmedly high, the role of IPF as an eventual marker of other autoimmune conditions is a field open to research.
- For both tests, padronization of normal levels and cutoff are a major need in order to facilitate interpretation of future studies.

## Author Contributions

DS, MP, TM, CF, and HS were equally responsible for the literature review, paper writing and reviewing the final document for submission. HS was responsible for project coordination and supervision.

## Conflict of Interest Statement

The authors declare that the research was conducted in the absence of any commercial or financial relationships that could be construed as a potential conflict of interest.
